# Alkaloids with Cardiovascular Effects from the Marine-Derived Fungus *Penicillium expansum* Y32

**DOI:** 10.3390/md13106489

**Published:** 2015-10-22

**Authors:** Ya-Qin Fan, Pei-Hai Li, Ya-Xi Chao, Hao Chen, Ning Du, Qiu-Xia He, Ke-Chun Liu

**Affiliations:** 1 Key Lab of Marine Bioactive Substances, First Institute of Oceanography, State Oceanic Administration, Qingdao 266061, China; E-Mails: fanyaqin.826@163.com (Y.-Q.F.); lipeihaihg@sina.com (P.-H.L.); yaxichao@163.com (Y.-X.C.); duning@fio.org.cn (N.D.); 2 Institute of Biology, Shandong Academy of Sciences; Research and Development Platform for Drug Screening of Shandong Academy of Sciences, Jinan 250014, China; E-Mails: heqiuxia8008@163.com (Q.-X.H.); hliukch@keylab.net (K.-C.L.)

**Keywords:** marine-derived fungus, *Penicillium expansum*, secondary metabolites, alkaloids, cardiovascular effects

## Abstract

Three new alkaloids (**1**, **4** and **8**), together with nine known analogues (**2**, **3**, **5**–**7**, and **9**–**12**), were isolated from the marine-derived fungus *Penicillium expansum* Y32. Their structures including the absolute configurations were elucidated by spectroscopic and Mosher’s and Marfey’s methods, along with quantum electronic circular dichroism (ECD) calculations. Each of the compounds was evaluated for cardiovascular effects in a live zebrafish model. All of the compounds showed a significant mitigative effect on bradycardia caused by astemizole (ASM) in the heart rate experiments. Compounds **4**–**6** and **8**–**12** exhibited potent vasculogenetic activity in vasculogenesis experiments. This is the first study to report that these types of compounds show cardiovascular effects in zebrafish. The results suggest that these compounds could be promising candidates for cardiovascular disease lead compounds.

## 1. Introduction

**Figure 1:**
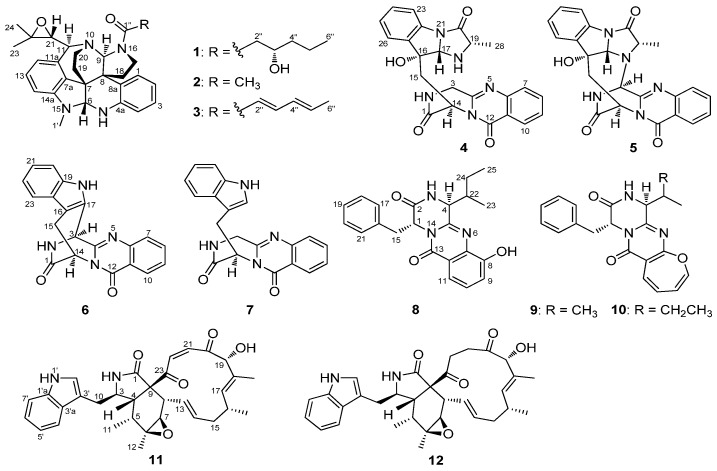
Structures of Compounds **1**–**12**.

According to the World Health Organization, cardiovascular diseases (CVDs) are the number one cause of death globally: more people die annually from CVDs than from any other cause. In 2012, 17.5 million people, representing 31% of all global deaths, died from this disease. CVDs are disorders of the heart and blood vessels and include coronary heart disease, cerebrovascular disease, peripheral arterial disease, rheumatic heart disease, congenital heart disease and other conditions [1]. Unfortunately, the number of people suffering from CVDs is on the rise, but only few and expensive drugs are available to treat the diseases. It is of great significance to search new and effective drugs to combat CVDs. In order to discover related lead compounds from marine-derived fungi, a fungus *Penicillium expansum* Y32 has been isolated from a seawater sample collected from the Indian Ocean. A chemical investigation of the ethyl acetate extract of fermentation broth of Y32 led to the identification of three new alkaloids, named communesin I (**1**), fumiquinazoline Q (**4**) and protuboxepin E (**8**), along with nine known analogues, communesin A and B (**2** and **3**) [2,3,4], cottoquinazoline A (**5**) [5], prelapatin B (**6**), glyantrypine (**7**) [6], protuboxepin A and B (**9** and **10**) [7], chaetoglobosin C (**11**) [8] and penochalasin E (**12**) [9] (Figure 1).

Compounds **1**–**12** were screened for cardiovascular effects in a model of live zebrafish, which provides a system for drug screening that combines the biological complexity of *in vivo* models with the ability for much higher throughput screening than other available animal models [10]. In the heart rate experiments, all of the compounds showed significant mitigative effects on bradycardia caused by astemizole (ASM) at different concentrations. In the vasculogenesis experiments, Compounds **4**–**6** and **8**–**12** (at concentrations of 20 μg/mL, 50 μg/mL and 100 μg/mL) exhibited potent vasculogenetic activity regarding vessel numbers, and four hits (**4**, **6**, **8** and **10**) displayed remarkable promoting functions regarding vessel length. Additionally, 12 compounds were also evaluated in antiangiogenic experiments, and no obvious activity was observed (the results are not listed). The results indicated that these compounds might be used for screening for new natural cardiovascular effect candidates.

## 2. Results and Discussion

**Figure 2:**
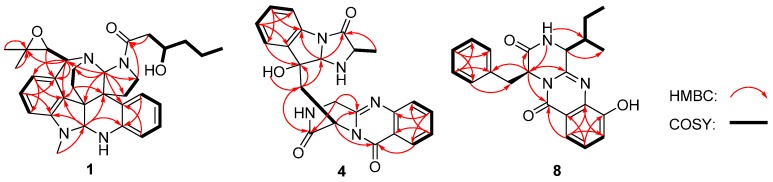
Key heteronuclear multiple-bond correlation spectroscopy (HMBC) and correlation spectroscopy (COSY) correlations of **1**, **4** and **8**.

Communesin I (**1**) was obtained as a white amorphous powder. The molecular formula was determined to be C_32_H_40_N_4_O_3_ using high-resolution electrospray ionisation mass spectrometry (HRESIMS) with a peak at *m*/*z* 529.3184 [M + H]^+^, indicating 15 degrees of unsaturation. The UV spectrum of **1** showed identical absorptions with communesin analogues at 206, 248, 268 and 320 nm [3]. The observed signals in ^1^H nuclear magnetic resonance (NMR), ^13^C NMR and DEPT spectra of **1** (Table 1) for twelve aromatic carbons (seven methines and five quaternary carbons) with seven protons (three triplets and four doublets) were indicative of a ortho-disubstituted benzene ring system and a 1,2,3-trisubstituted benzene system. Those deductions were further verified by the key correlation spectroscopy (COSY) correlations of H-1 (δ_H_ 6.68, d, *J* = 7.6 Hz)/H-2 (δ_H_ 6.71, t, *J* = 7.6 Hz)/H-3 (δ_H_ 7.01, t, *J* = 7.6 Hz)/H-4 (δ_H_ 6.69, d, *J* = 7.6 Hz) and H-12 (δ_H_ 6.05, d, *J* = 7.7 Hz)/H-13 (δ_H_ 6.88, t, *J* = 7.7 Hz)/H-14 (δ_H_ 5.95, d, *J* = 7.7 Hz) along with the heteronuclear multiple-bond correlation spectroscopy (HMBC) correlations from H-1 and H-3 to the quaternary carbon C-4a (δ_C_ 142.6), from H-2 and H-4 to the quaternary carbon C-8a (δ_C_ 132.1), from H-12 and H-14 to the quaternary carbon C-7a (δ_C_ 132.1) and from H-13 to two quaternary carbons (C-11a, δ_C_ 136.1; C-14a, δ_C_ 150.5). The resonances at δ_C_ 29.6 (qC) and δ_H_ 2.84 (3H, s) indicated the presence of an *N*-methyl group. In addition, three methyls, seven methylenes, five methines and four quaternary carbons appeared in the ^1^H NMR, ^13^C NMR, and DEPT spectra. Combined with the COSY spectrum, two –CH_2_–CH_2_– spin systems, one 2,2-dimethyloxirane group and one pentan-2-ol moiety were deduced (Figure 2). Extensive comparison of the ^1^H and ^13^C NMR spectra with those of communesin A (**2**) revealed that the structures of these two compounds were very similar, except for the fact that the signals of the methyl group (C-2″) in communesin A were replaced by the pentan-2-ol moiety in **1.** The assignment was confirmed by the HMBC correlations from H-6 (δ 4.70) to C-4a (δ 142.6), C-7a (δ 132.1), C-8 (δ 52.1) and C-14a (δ 150.5), from H_3_-1′ (δ 2.84) to C-6 (δ 82.4) and C-14a (δ 150.5), from H-9 (δ 5.05) to C-7 (δ 51.3), C-8a (δ 132.1), C-11 (δ 65.2), C-17(δ 44.1), C-18 (δ 30.6) and C-20 (δ 36.1), from H-11 (δ 4.11) to C-7a (δ 132.1), C-9 (δ 79.1), C-12 (δ 113.1) and C-20, from H_b_-17 (δ 3.00) to C-1″ (δ 173.9), from H_2_-18 (δ 2.71/2.00) to C-7 (δ 51.3) and C-8a (δ 132.1), from H_2_-19 (δ 2.37/2.28) to C-6 (δ 82.4), C-7a (δ 132.1 ) and C-8 (δ 52.1), and from H_2_-2″ (δ 2.82/2.48) to C-1″ (δ 173.9) (Figure 2). Therefore, the planar structure of communesin I (**1**) was established.

**Figure 3:**
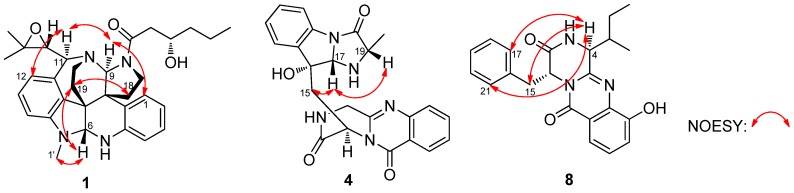
Key nuclear Overhauser effect spectroscopy (NOESY) correlations of **1**, **4** and **8**.

**Figure 4:**
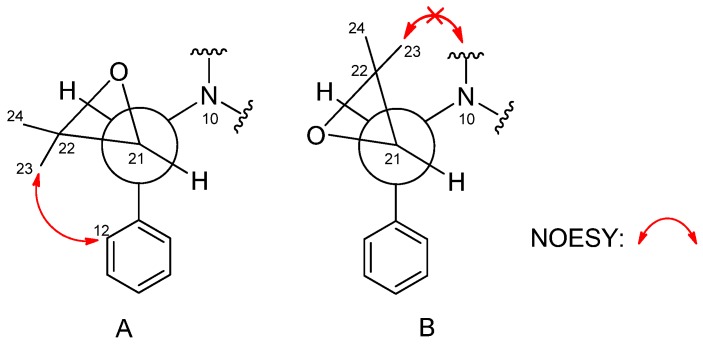
Stereochemistry of C-21 and C-11 in Compound **1** (*J*_21,__11_ = 9.0 Hz, large).

The relative configuration was determined by nuclear Overhauser effect spectroscopy (NOESY) experiments and Murata’s *J*-based method [3,11]. The NOESY correlations of H-1/H-9 (δ_H_ 5.05)/H-11 (δ_H_ 4.11) /H-12 (δ_H_ 6.05), H_3_-1′ (δ_H_ 2.84)/H-6 (δ_H_ 4.70)/H_2_-19 (δ 2.37/2.28), and H_a_-19 (δ_H_ 2.37)/H_a_-18 (δ_H_ 2.71) confirmed the two –CH_2_–CH_2_– bridges, H-6 and H_3_-1″, to be on the same side, which displayed a similar correlative pattern to those of **2** (Figure 3). Application of Murata’s *J*_H-H_-based method [3] enabled to determine the relative stereochemistry at C-21. The large coupling constant between H-21 and H-11 (*J*_H-H_ = 9 Hz) indicated an approximate 180° (anti arrangement) of the dihedral angles of H11-C11-C21-H21 (Figure 4). Additionally, the NOE correlations of H_3_-23/H_3_-24/H-12 and the absent correlations of the geminal methyl groups to H-9 or H_2_-19/20 supported the theory that the epoxide oxygen was oriented syn to *N*-10 as shown in conformation A in Figure 4.

**Table 1:** ^1^H and ^13^C nuclear magnetic resonance (NMR) data of Compounds **1** and **8**.

1 ^a^			8 ^b^		
Position	δ_C_	δ_H_, Mult. (*J* in Hz)	Position	δ_C_	δ_H_, Mult. (*J* in Hz)
1	123.2, CH	6.68, d (7.6)	1	56.5, CH	5.29, t (5.0)
2	120.6, CH	6.71, t (7.6)	2	167.4, C	
3	127.5, CH	7.01, t (7.6)	3		8.32, brs
4	117.0, CH	6.69, d (7.6)	4	57.8, CH	3.19, d (1.7)
4a	142.6, C		5	149.1, C	
6	82.4, CH	4.70, s	7	135.7, C	
7	51.3, C		8	152.6, C	
7a	132.1, C		9	118.7, CH	7.25, d (7.9)
8	52.1, C		10	127.3, CH	7.37, t ( 7.9)
8a	132.1, C		11	116.0, CH	7.59, d (7.9)
9	79.1, CH	5.05, s	12	120.5, C	
11	65.2, CH	4.11, d (9.0)	13	160.0, C	
11a	136.1, C		15	36.4, CH_2_	3.27, dd (9.2, 5.0) 3.32 ^c^, m
12	113.1, CH	6.05, d (7.7)	16	135.4, C	
13	129.0, CH	6.88, t (7.7)	17	129.5, CH	6.92, d (7.0)
14	101.9, CH	5.95, d (7.7)	18	128.5, CH	7.20, t (7.0)
14a	150.5, C		19	127.5, CH	7.24, m
17a	44.1, CH_2_	3.92, dd (12.2, 8.7); 3.00, dt (12.2, 7.1)	20	128.5, CH	7.20, t (7.0)
17b					
18a	30.6, CH_2_	2.71, dt (12.2, 8.7); 2.00, dd (12.2, 7.1)	21	129.5, CH	6.92, d (7.0)
18b					
19a	37.7, CH_2_	2.37, dd (12.8, 8.5); 2.28, dt (12.8, 9.5)	22	35.0, CH	2.69, m
19b					
20a	36.1, CH_2_	3.46, dd (15.8, 9.5); 3.36, dt (15.8, 8.5)	23	15.4, CH_3_	0.91, d (7.3)
20b					
21	64.1, CH	2.87, d (9.0)	24	22.8, CH_2_	1.17, m
22	60.4, C		25	12.2, CH_3_	0.73, t (7.4)
23	24.9, CH_3_	1.39, s	8-OH		9.50, s
24	20.3, CH_3_	1.57, s			
1ʹ	29.6, CH_3_	2.84, s			
1″	173.9, C				
2″	42.1, CH_2_	2.82, dd (14.6, 3.4); 2.48, dd (14.6, 3.4)			
3″	69.0, CH	4.06, brs			
4″	39.5, CH_2_	1.60, m; 1.53, m			
5ʹʹ	18.9, CH_2_	1.54, m; 1.44, m			
6ʹʹ	14.1, CH_3_	0.96, t (7.0)			
OH		4.14, brs			

^a^ Measured in CDCl_3_ (^1^H 600 MHz, ^13^C 150 MHz, TMS, δ ppm); ^b^ Measured in DMSO-*d*_6_ (^1^H 600 MHz, ^13^C 150 MHz, TMS, δ ppm); ^c^ Overlapped with H_2_O signal in DMSO-*d*_6_.

**Figure 5:**
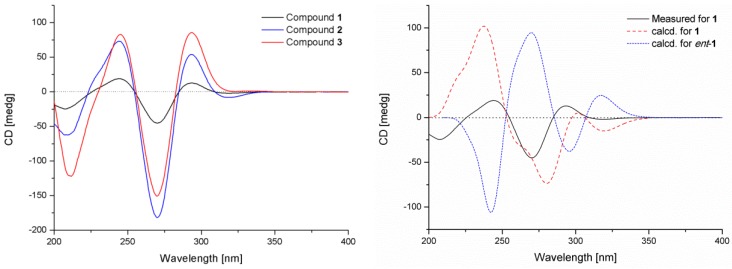
Measured CD curves of **1**–**3** and calculated equivalent circulating density (ECD) curves of **1** and *ent*-**1**.

Compound **1** displayed a similar CD Cotton effect (Figure 5) to those of communesin A (**2**) and B (**3**) (Figure 5), absolute configurations of which have been reported in the previous literature [4], indicating the same 6*R*, 7*R*, 8*R*, 9*S*, 11*S* and 21*R* configurations. This was further confirmed by quantum chemical equivalent circulating density (ECD) calculation of **1** and *ent*-**1** using Gaussian 09 (Figure 5) [12]. The preliminary conformational distribution search was performed using the HyperChem 7.5 software. The corresponding minimum geometries were further fully optimized using the density functional theory (DFT) at the B3LYP/6-31G(d) level as implemented in the Gaussian 09 program package [12]. The stable conformers obtained were submitted to ECD calculation using the time-dependent DFT (TDDFT) method (B3LYP/6-31G(d)). The overall predicted ECD spectrum of **1** was subsequently compared with the measured one. Finally, the measured CD curve matched well with the calculated curve for **1** and was opposite to that of *ent*-1 (Figure 5).

**Figure 6:**
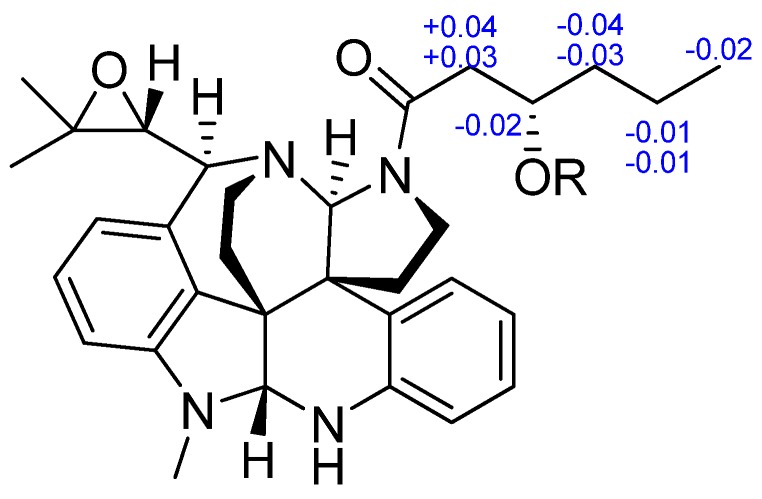
Δδ (= δ*_S_* − δ*_R_*) values for (*S*)- and (*R*)-MTPA esters of **1**.

The absolute configuration at C-3″ was determined using the modified Mosher’s method [13]. Compound **1** esterified was separately with (*R*)-MTPA and (*S*)-MTPA chloride to give the (*S*)-MTPA and (*R*)-MTPA esters, **1a** and **1b**, respectively. The distribution of Δδ values between **1a** and **1b** clearly defined the *S*-configuration at C-3″ (Figure 6). Thus, the new Structure **1** was established as (6*R*, 7*R*, 8*R*, 9*S*, 11*S*, 21*R* and 3″*S*).

**Figure 7:**
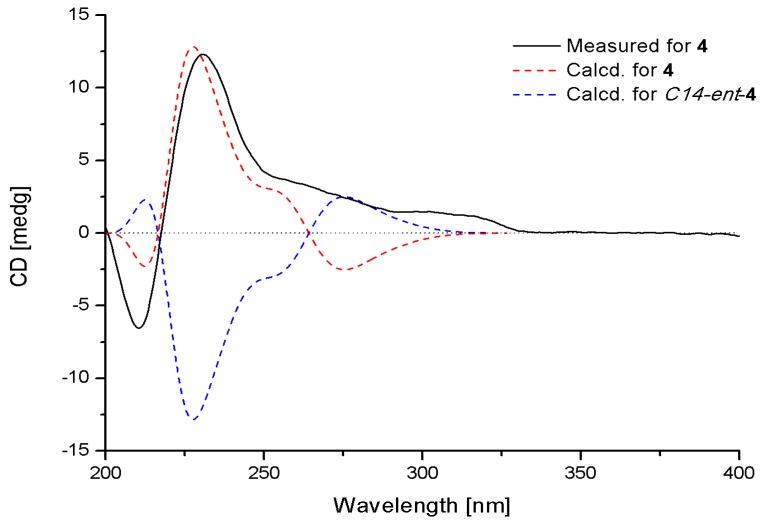
Measured and calculated ECD spectra for **4**.

Fumiquinazoline Q (**4**) was obtained as a white amorphous powder. The molecular formula C_23_H_21_N_5_O_4_ was assigned on the basis of the HRESIMS peak at *m*/*z* 432.1673 [M + H]^+^ (calcd. 432.1672), requiring 16 degrees of unsaturation. The IR absorption bands at 3436, 1685 and 1611 cm^−1^ of **4** suggested the presence of hydroxy and amido-carbonyl groups [14]. In the ^1^H NMR spectrum, the signals for two ortho-disubstituted benzene ring system were present at δ_H_ 7.63 (1H, d, *J* = 8.0 Hz, H-7)/δ_H_ 7.78 (1H, t, *J* = 8.0 Hz, H-8)/δ_H_ 7.50 (1H, t, *J* = 8.0 Hz, H-9)/δ_H_ 8.30 (1H, d, *J* = 8.0 Hz, H-10) and δ_H_ 7.52 (1H, d, *J* = 7.4 Hz, H-23)/δ_H_ 7.08 (1H, t, *J* = 7.4 Hz, H-24)/δ_H_ 7.30 (1H, t, *J* = 7.4 Hz, H-25)/δ_H_ 7.31 (1H, d, *J* = 7.4 Hz, H-26), which were further confirmed by COSY (Figure 2). The ^13^C NMR data (Table 2) revealed the presence of 23 carbon signals, sorted by DEPT experiments into one methyl, two methylenes, eleven methines including eight aromatic methines, and nine quaternary carbons including two conjugated carbonyl groups (δ_C_ 160.4, C-12; 172.9, C-20). ^1^H and ^13^C NMR (Table 2) spectra analysis revealed that **4** was an analogue of the known fumiquinazoline A [15], in which a methine (δ_C/H_ 49.2/4.88) was replaced by a methylene signal (δ_C/H_ 45.3/4.70, 4.49) in **4**, whereas a methyl (δ_C/H_ 16.8/1.79) in fumiquinazolines A was absent. These data were further confirmed by the heteronuclear single quantum coherence (HSQC) of H_a_-3 (δ_H_ 4.70) and H_b_-3 (δ_H_ 4.49) to C-3 (δ_C_ 45.3), the ^1^H-^1^H COSY of H_b_-3/NH-2 (δ_H_ 7.01) along with HMBC (Figure 2) correlations from H_2_-3 (δ_H_ 4.70/4.49) to C-4 (δ_C_ 148.0) and C-1 (δ_C_ 169.9). Thus, the planar structure of **4** was established as shown in Figure 2. The relative configuration of **4** could be deduced from the NOESY data (Figure 3). The NOESY correlations of H_a_-15 (δ 2.37)/H-17 (δ 5.25)/H-19 (δ 4.25) suggested that H-17/H-19 was in *cis*-configuration and H-17/OH-16 was in *trans*-configuration. The absolute configuration at C-19 was confirmed by acidic hydrolysis of **4**, which afforded l-Ala that was determined by Marfey’s method [16]. The absolute configuration at C-14 was also determined by ECD calculations of **4** and *ent*-**4** [12]. The result showed that CD curve of **4** was consistent with the calculated ECD curve of **4** but opposite to that of *ent*-**4** (Figure 7). Therefore, the absolute configuration of **4** was unambiguously assigned as (14*R*, 16*R*, 17*S*, 19*S*).

**Table 2:** ^1^H and ^13^C NMR Data of Compounds **4** and **5**.

Position	4 ^a^		5 ^a^	
	δ_C_	δ_H_, Mult. (*J* in Hz)	δ_C_	δ_H_, Mult. (*J* in Hz)
1	169.9, C		170.1, C	
2		7.01, brs		8.26, brs
3a	45.3, CH_2_	4.70, d (15.6)	66.2, CH	5.31, d (3.1)
3b		4.49, d (15.6)		
4	148.0, C		146.8, C	
6	147.1, C		146.4, C	
7	127.1, CH	7.63, d (8.0)	127.8, CH	7.64, d (7.9)
8	135.1, CH	7.78, t (8.0)	135.3, CH	7.72, t (7.9)
9	127.6, CH	7.50, t (8.0)	127.0, CH	7.45, t (7.9)
10	127.3, CH	8.30, d (8.0)	125.6, CH	8.18, d (7.9)
11	120.5, C		120.5, C	
12	160.4, C		159.8, C	
14	51.5, CH	5.81, dd (8.6, 3.8)	54.1, CH	5.62, dd (4.7, 2.3)
15a	39.7, CH_2_	2.37, dd (14.5, 8.9)	37.2, CH_2_	2.49, dd (15.3, 2.1)
15b		2.74, dd (14.5, 3.9)		3.28, dd (15.3, 5.1)
16	74.2, C		74.8, C	
17	79.8, CH	5.25, s	80.9, CH	4.58, d (1.3)
19	59.0, CH	4.25, q (12.8, 6.2)	64.3, CH	4.21, q (11.9, 6.5)
20	172.9, C		165.8, C	
22	137.4, C		136.1, C	
23	115.8, CH	7.52, d (7.4)	115.2, CH	7.39, d (7.6)
24	125.3, CH	7.08, t (7.4)	128.1, CH	7.24, t (7.6)
25	130.3, CH	7.30, t (7.4)	130.5, CH	7.08, t (7.6)
26	124.2, CH	7.31, d (7.4)	124.4, CH	7.34, d (7.6)
27	138.0, C		138.1, C	
28	18.2, CH_3_	1.39, d (6.6)	14.9, CH_3_	1.51, d (6.5)
16-OH		5.00, brs		4.64, brs

^a^ Measured in CDCl_3_ (^1^H 600 MHz, ^13^C 150 MHz, TMS, δ ppm).

Protuboxepin E (**8**) was obtained as a white amorphous powder. The molecular formula of **8** was determined to be C_22_H_23_N_3_O_3_ by analysis of ^1^H, ^13^C and DEPT NMR data and was verified by the HRESIMS peak at *m*/*z* 378.1821 [M + H]^+^ (calcd. 378.1818), indicating 13 degrees of unsaturation. Its IR spectrum exhibited absorption at 3610, 1679 and 1539 cm^−1^, characteristic of hydroxyl, amide and aromatic ring groups [17]. The ^1^H NMR spectrum of **8** showed the signals for a 1,2,3-trisubstituted benzene ring system, and a monosubstituted benzene ring system was present at δ_H_ 7.25 (1H, d, *J* = 7.9 Hz, H-9)/δ_H_ 7.37 (1H, t, *J* = 7.9 Hz, H-10)/δ_H_ 7.59 (1H, d, *J* = 7.9 Hz, H-11) and δ_H_ 6.92 (2H, d, *J* = 7.0 Hz, H-17/21)/δ_H_ 7.20 (2H, t, *J* = 7.0 Hz, H-18/22)/δ_H_ 7.24 (1H, m, H-19), which was further confirmed by COSY (Figure 2). The ^13^C NMR data (Table 1) revealed the presence of 22 carbon signals, sorted by DEPT experiments into two methyls, two methylenes, eleven methines including eight aromatic methines, and seven quaternary carbons including two conjugated carbonyl groups (δ_C_ 167.4, C-2; 160.0, C-13). The NMR data of **8** implied that it was an analogue of protuboxepin A [7], except for the obvious upfielded shift for C-7 and downfielded shifts for C-8 and C-12 in **8**. Meanwhile, C-9 in protuboxepin A had been transformed from a methine into a quaternary carbon in **8**, which implied a replacement of the 7-membered ring A in protuboxepin A by a hexatomic benzene ring in **8** with the ether bond in protuboxepin A changed to a hydroxyl in **8**. The ^1^H-^1^H COSY experiment exhibited correlations of H-4 (δ_H_ 3.19)/H-22 (δ_H_ 2.69)/H_2_-24 (δ_H_ 1.17)/H_3_-25 (δ_H_ 0.73) (Figure 2), the HMBC experiment showed correlations from H-9 (δ_H_ 7.25) to C-7 (δ_C_ 135.7) and C-11 (δ_C_ 116.0), H-10 (δ_H_ 7.37) to C-8 (δ_C_ 152.6) and C-12 (δ_C_ 120.5), H-11 (δ_H_ 7.59) to C-7 (δ_C_ 135.7), C-9 (δ_C_ 118.7) and C-13 (δ_C_ 160.0), H_3_-25 to C-22 (δ_C_ 35.0) and H_2_-24 to C-4 (δ_C_ 57.8). These data further proved the planar construction of **8** shown in Figure 1. The observation of key NOESY correlations of H-4 with H_2_-15, H-17 and H-21 indicated a *trans*-orientation of H-1/H-4. In addition, Marfey’s analysis of the acid hydrolysis of **8** afforded a d-phenylalanine residue. Therefore, the absolute configurations of C-1 and C-4 were determined as *R* and *S*, respectively. Murata’s *J*-based method and Marfey’s analysis were applied to assign the absolute stereochemistry at C-22, but it was unfortunately unsuccessful because of the uncertainty of the single bond C4-C22, which could rotate freely. Thus, the configuration of C-22 has not been determined.

The partial absolute stereostructure of the known Compound **5** was first determined by the Marfey’s method [5]. According to the l-Ala residue in acidic hydrolysis of **5**, the partial absolute stereostructure of **5** was unambiguously assigned as (16*R*, 17*S*, 19*S*).

**Figure 8:**
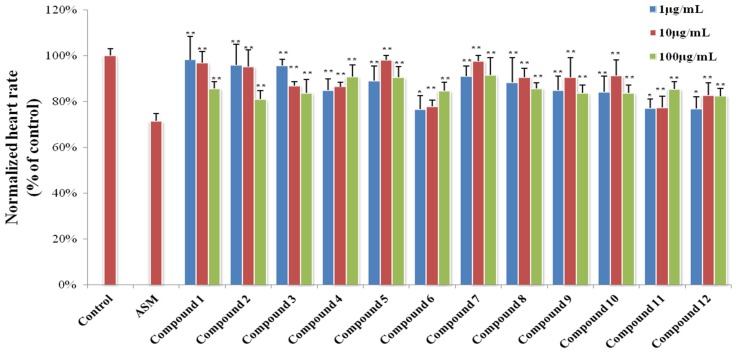
Results of zebrafish heart rate experiments. Significant difference between compound treatment and Astemizole (ASM) (* *p* < 0.05; ** *p* < 0.01). The 1‰ DMSO-treated group is represented as “Control”.

**Figure 9:**
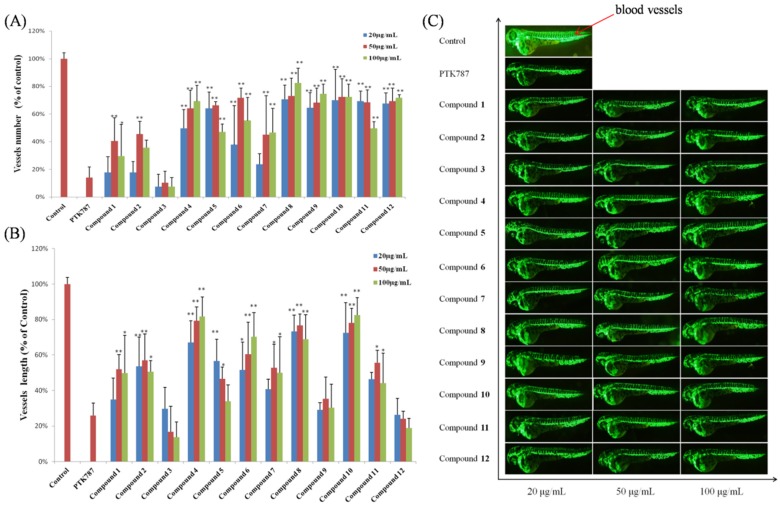
Results of vasculogenesis experiments. (**A**) Number of vessels in zebrafish treated with 12 compounds in the vasculogenesis experiments; (**B**) vessel length of zebrafish treated with 12 compounds in the vasculogenesis experiments; (**C**) representative images of zebrafish treated with 12 compounds in the vasculogenesis experiments (blood vessels are shown as red arrows). Significant difference between compound treatment and PTK787 (* *p* < 0.05; ** *p* < 0.01). The 1‰ DMSO-treated group is represented as “Control”.

Zebrafish embryos of the AB wild-type strain and TG (VEGFR2: GFP) type strain with fluorescent blood vessels were used to screen Compounds **1**–**12** for cardiovascular effects [18,19,20]. In the heart rate experiments, all of the compounds showed a significant mitigative effect on bradycardia caused by astemizole (ASM) at different concentrations (Figure 8). Figure 9 gives representative examples of the vasculogenetic effect observed in zebrafish by the test compounds. Regarding number of vessels, Compounds **4**–**6** and **8**–**12** (at concentrations of 20 μg/mL, 50 μg/mL and 100 μg/mL) exhibited potent vasculogenetic activity and Compounds **1**, **2** and **7** had moderate effects, while Compound **3** was ineffective. Regarding vessels length, four hits (**4**, **6**, **8** and **10**) displayed remarkable promoting function; mild effects were identified for Compounds **1**, **2**, **5**, **7** and **11**. Compounds **3**, **9** and **12** did not show any relevant activity. As additional investigations aiming at screening for cardiovascular effects in the model of live zebrafish, 12 compounds were also evaluated in an antiangiogenic experiment, and no obvious activity was observed. This is the first report showing cardiovascular effects of these compounds in zebrafish.

## 3. Experimental Section

### 3.1. General Experimental Procedures

Optical rotations were measured on a Perkin-Elmer 341 digital polarimeter (Perkin-Elmer, Waltham, MA, USA). UV spectra were recorded on an eppendorf BioSpectrometer Basic photometer. IR spectra were obtained on a Nicolet Nexus 470 spectropolarimeter (Thermo Nicolet Analytical Instruments, Madison, WI, USA) as KBr discs. CD data were measured on a JASCO J-715 spectropolarimeter (Jasco, Tokyo, Japan). ^1^H NMR, ^13^C NMR, DEPT, HMQC, HMBC, COSY and NOESY spectra were recorded using a JEOL JNM-ECP 600 spectrometer (JEOL, Tokyo, Japan). HRESIMS spectra were measured on an Agilent Technologies G1969A mass spectrometer (Agilent Technologies Inc., Santa Clara, CA, USA). Analytical high performance liquid chromatography (HPLC) system (HITACHI, Tokyo, Japan) consisted of Organizer, UV Detector L-2400, Pump L-2130 and software Hitachi Model D-2000 Elite using a C18 column (YMC-pack ODS-A, 150 × 4.6 mm, S-5 μM, 12 nm, 1 mL/min). Semipreparative HPLC was operated on the same system using a Prep RP-18 column (YMC-pack ODS-A, 250 × 10 mm, S-5 μM, 12 nm, 2.5 mL/min) with UV detection. Vacuum-liquid chromatography (VLC) used silica gel H (Qingdao Marine Chemical Factory, Qingdao, China). Thin layer chromatography (TLC) and column chromatography were performed on plates pre-coated with silica gel GF254 (10–40 μm) and Sephadex LH-20 (Amersham Biosciences, Piscataway, NJ, USA), respectively.

### 3.2. Fungal Material and Fermentation

The fungus *Penicillium expansum* Y32 was originally obtained from a seawater sample collected from a depth of about 30 m in the Indian Ocean (88°59′51″ E, 2°59′54″ S) in 2013. The sample was cultured in a Malt Extract Agar (MEA: Malt extract, 17 g; Peptone, 3 g; Agar, 20 g; sea water, 1 L) plate using chloramphenicol (100 μg/mL) as a bacterial inhibitor. A single colony was transferred onto another MEA plate and was identified according to its morphological characteristics and 18S rRNA gene sequences (GenBank access No. KP872504, supplementary materials). The fungus *Penicillium expansum* Y32 was cultured in 1000 mL conical flasks containing 300 mL fermentation media consisting of 1.7% malt extract and 0.3% peptone at 28 °C for 30 days.

### 3.3. Extraction and Purification

The whole fermentation broth (15 L) of cultivated medium was extracted exhaustively with EtOAc, while the mycelia were extracted with 80% volume aqueous acetone. After removing the acetone by evaporation under vacuum, the obtained aqueous acetone solution was extracted three times with EtOAc. The combined EtOAc extracts were dried under vacuum to produce 10.1 g of extract. The combined extract was subjected to silica gel chromatography using a vacuum liquid chromatography (VLC) column, eluting with a stepwise gradient of a mixture of petroleum ether (PE)-CH_2_Cl_2_ (2:1 and 0:1), then of CH_2_Cl_2_-MeOH (100:1, 50:1, 30:1, 10:1, 1:1, and 0:1) to yield eight major primary fractions (Fr.1–Fr.10). Fraction 6 was eluted with CH_2_Cl_2_-MeOH (100:1) and was further resolved into four fractions (Fr.6.1–Fr.6.4) followed by reversed-phase C18 silica column chromatography eluting with a stepwise gradient of 30% to 100% MeOH in H_2_O. Fraction 3 (72 mg) was eluted with CH_2_Cl_2_-MeOH (60:1) and was further resolved into four fractions (Fr.3.1–Fr.3.4) followed by reversed-phase C18 silica column chromatography (30 × 400 mm) eluting with a stepwise gradient of 30% to 90% MeOH in H_2_O. Fraction 3.4 (90% MeOH) was further purified by Sephadex LH-20 chromatography (20 × 1000 mm) eluting with MeOH to obtain Fr. 3.4.1–Fr. 3.4.3. Fraction 3.4.1 was then further purified by semi-preparative HPLC over ODS (70% MeOH-H_2_O, *v*/*v*) to give Compound **1** (*t*_R_ = 23.3 min; 1.7 mg) and compound **2** (*t*_R_ = 18.6 min, 11.3 mg). Fraction 3.4.2 was subjected to HPLC over ODS (70% MeOH-H_2_O, *v*/*v*) to yield Compound **3** (*t*_R_ = 29.0 min, 30 mg). Fraction 4 (123 mg) was subjected to reversed-phase C18 silica column chromatography (30 × 400 mm) eluting with CH_2_Cl_2_/MeOH (10%–90%) to afford five subfractions (Fr.4.1–Fr.4.5). Fraction 4.3 was separated by Sephadex LH-20 eluting with MeOH to provide five subfractions (Fr.4.3.1–Fr.4.3.5). Compounds **4** (*t*_R_ = 15.9 min, 1.8 mg) and **5** (*t*_R_ = 11.3 min, 7.1 mg) were purified from Fr.4.3.2 and Fr.4.3.3 by semi-preparative HPLC eluting with 70% and 60% MeOH-H_2_O, respectively. Fraction 4.4 was purified by HPLC on ODS (70% MeOH-H_2_O, *v*/*v*) to yield **6** (*t*_R_ = 11.5 min, 1.1 mg) and **7** (*t*_R_ = 10.5 min, 5.1 mg). Fraction 6 (52 mg) was separated by Sephadex LH-20 column (20 × 1000 mm), eluting with MeOH, to afford three subfractions (Fr.6.1–Fr.6.3). Fraction 6.1 was further purified by HPLC over ODS (70% MeOH-H_2_O, *v*/*v*) to afford **8** (*t*_R_ = 13.8 min, 2.7 mg) and **9** (*t*_R_ = 15.9 min, 1.5 mg). Compound **10** (*t*_R_ 10.4 min, 1.2 mg) was crystallized from a 70% MeOH-H_2_O solution of subfraction 6.3. Fraction 2 (151 mg) was eluted with a stepwise gradient of 30% to 90% MeOH in H_2_O by reversed-phase C18 silica column chromatography (30 × 400 mm). Fraction 2.4 (90% MeOH) was further purified by semi-preparative HPLC over ODS (70% MeOH-H_2_O, *v*/*v*) to give Compounds **11** (*t*_R_ = 14.1 min, 5.97 mg) and **12** (*t*_R_ = 22.4 min, 48.3 mg).

*Communesin I* (**1**)*:* White amorphous powder; ${\text{[α]}}_{\text{D}}^{20}$ –59 (*c* 0.1, MeOH); UV (MeOH) λ_max_ 206 (2.16), 248 (2.39), 268 (2.32), 320 (1.86) nm; CD (*c* 0.1, MeOH) λ_max_ (Δε) 208 (−1.2), 244 (+0.8), 270 (−1.5), 294 (+0.9), 319 (−0.1) nm; IR (KBr) ν_max_ 3618, 2926, 1740, 1692, 1646, 1548, 1532, 1512, 1427, 1390, 1339, 1012, 754 cm^−1^; ^1^H and ^13^C NMR data, see Table 1; HRESIMS *m*/*z* 529.3184 [M + H]^+^ (calcd for C_32_H_41_N_4_O_3_, 529.3179).

*Fumiquinazoline Q* (**4**)*:* White amorphous powder; ${\text{[α]}}_{\text{D}}^{20}$ +50 (*c* 0.1, MeOH); UV (MeOH) λ_max_ 204 (2.49), 227 (2.36) and 304 (1.46) nm; CD (*c* 0.1, MeOH) λ_max_ (Δε) 210 (−0.4), 230 (+0.7), 302 (+0.1) nm; IR (KBr) ν_max_ 3745, 3436, 2925, 1685, 1611, 1480, 1389, 1339, 1256, 1025, 769 cm^−1^; ^1^H and ^13^C NMR data, see Table 2; HRESIMS *m*/*z* 432.1673 [M + H]^+^ (calcd for C_23_H_22_N_5_O_4_, 432.1672).

*Cottoquinazoline A* (**5**)*:* White amorphous powder; ${\text{[α]}}_{\text{D}}^{20}$ +80 (*c* 0.1, MeOH); UV (MeOH) λ_max_ 225 (2.32), 250 (1.33), 303 (0.10) nm; ^1^H and ^13^C NMR data, see Table 2; HRESIMS *m*/*z* 430.1499 [M + H]^+^ (calcd for C_23_H_20_N_5_O_4_, 430.1510).

*Protuboxepin E* (**8**)*:* White amorphous powder; ${\text{[α]}}_{\text{D}}^{20}$ −57 (*c* 0.1, MeOH); UV (MeOH) λ_max_ 237 (2.63), 285 (2.16), 321 (2.15) nm; CD (*c* 0.1, MeOH) λ_max_ (Δε) 238 (−1.1), 326 (−0.5) nm; IR (KBr) ν_max_ 3672, 3610, 2968, 1679, 1539, 1514, 1456, 1389, 1215, 762 cm^−1^; ^1^H and ^13^C NMR data, see Table 1; HRESIMS *m*/*z* 378.1821 [M + H]^+^ (calcd for C_22_H_24_N_3_O_3_, 378.1818).

### 3.4. Preparation of the (S)-and (R)-MTPA Esters of **1a** and **1b** by Modified Mosher’s Method

Compound **1** (1 mg for each) and 4-dimethylaminopyridine (1 mg) were dissolved with anhydrous CH_2_Cl_2_ (500 μL). Under N_2_ atmosphere, triethylamine (20 μL) and *R*-(−)- or *S*-(+)-MTPA chloride (10 μL) were added. The reaction mixture was stirred at room temperature for 2 h and quenched with saturated aqueous sodium bicarbonate (1 mL). Extraction with CH_2_Cl_2_ (3 × 5 mL) was carried out. The organic layers combined and separated by semipreparative HPLC on ODS (80% MeOH-H_2_O, *v*/*v*) to afford the *S*-MTPA ester **1a** (1.2 mg, *t*_R_ = 10.2 min) and *R*-MTPA ester **1b** (0.8 mg, *t*_R_ = 10.4 min), respectively.

*Compound **1a**:* ^1^H NMR (CDCl_3_, 600 MHz) δ 6.98 (t, *J* = 7.7 Hz, 1H, H-3), 6.85 (t, *J* = 7.7 Hz, 1H, H-13), 6.77 (d, *J* = 7.7 Hz, 1H, H-2), 6.66 (d, *J* = 7.7 Hz, 1H, H-4), 6.65 (d, *J* = 7.7 Hz, 1H, H-1), 6.02 (d, *J* = 7.7 Hz, 1H, H-12), 5.92 (d, *J* = 7.7 Hz, 1H, H-14), 5.74 (m, 1H, H-3″), 5.06 (s, 1H, H-9), 4.70 (s, 1H, H-6), 4.04 (d, *J* = 8.9 Hz, 1H, H-11), 3.84 (dd, *J* = 11.3, 8.8 Hz, 1H, H_a_-17), 3.41 (d, *J* = 16.6 Hz, 1H, H_a_-2″), 3.39 (dd, *J* = 16.2, 8.0 Hz, 1H, H_a_-20), 3.24 (dd, *J* = 16.2, 8.0 Hz, 1H, H_b_-20), 2.93 (dd, *J* = 11.4, 8.6 Hz, 1H, H- H_b_-17), 2.85 (s, 3H, H-1′), 2.83 (d, *J* = 8.9 Hz, 1H, H-21), 2.75 (dd, *J* = 16.6, 5.5 Hz, 1H, H_b_-2″), 2.71 (dd, *J* = 13.0, 7.0 Hz, 1H, H_a_-18), 2.34 (dt, *J* = 12.6, 8.8 Hz, 1H, H_a_-19), 2.24 (dt, *J* = 12.5, 8.6 Hz, 1H, H_b_-19), 1.97 (dd, *J* = 13.1, 6.9 Hz, 1H, H_b_-18), 1.70 (m, 1H, H_a_-4″), 1.68 (m, 1H, H_b_-4″), 1.44 (s, 3H, H_3_-24), 1.35 (s, 3H, H_3_-23), 1.30 (m, 1H, H_a_-5″), 1.25 (m, 1H, H_b_-5″), 0.90 (s, 3H, H_3_-6″); ESIMS *m/z* 745.4 [M + H]^+^.

*Compound **1b**:* ^1^H NMR (CDCl_3_, 600 MHz) δ 7.00 (t, *J* = 7.6 Hz, 1H, H-3), 6.88 (t, *J* = 7.7 Hz, 1H, H-13), 6.71 (d, *J* = 7.6 Hz, 1H, H-2), 6.69 (d, *J* = 7.6 Hz, 1H, H-4), 6.66 (d, *J* = 7.6 Hz, 1H, H-1), 6.05 (d, *J* = 7.7 Hz, 1H, H-12), 5.95 (d, *J* = 7.7 Hz, 1H, H-14), 5.76 (m, 1H, H-3″), 5.05 (s, 1H, H-9), 4.69 (s, 1H, H-6), 4.03 (d, *J* = 8.8 Hz, 1H, H-11), 3.90 (dd, *J* = 11.8, 8.6 Hz, 1H, H_a_-17), 3.42 (m, H_a_-20), 3.37 (dd, *J* = 16.3, 6.0 Hz, 1H, H_a_-2″), 3.27 (m, 1H, H_b_-20), 2.99 (dd, *J* = 11.7, 8.6 Hz, 1H, H- H_b_-17), 2.84 (s, 3H, H-1′), 2.83 (d, *J* = 9.0 Hz, 1H, H-21), 2.72 (dd, *J* = 16.5, 5.9 Hz, 1H, H_b_-2″), 2.69 (dd, *J* = 13.1, 7.1 Hz, 1H, H_a_-18), 2.33 (dd, *J* = 12.0, 8.7 Hz, 1H, H_a_-19), 2.25 (m, 1H, H_b_-19), 1.97 (dd, *J* = 13.2, 6.9 Hz, 1H, H_b_-18), 1.74 (m, 1H, H_a_-4″), 1.71 (m, 1H, H_b_-4″), 1.41 (s, 3H, H_3_-24), 1.34 (s, 3H, H_3_-23), 1.31 (m, 1H, H_a_-5″), 1.26 (m, 1H, H_b_-5″), 0.92 (s, 3H, H_3_-6″); ESIMS *m/z* 745.4 [M + H]^+^.

### 3.5. Marfey’s Analysis

A solution of Compound **4** (0.5 mg)/Compound **5** (0.5 mg) in 6 M HCl (0.5 mL) was heated at 110 °C for 19 h. Then the solution was evaporated to dryness. The residue, l-Ala and d-Ala were dissolved in H_2_O (250 μL each), respectively. One hundred microliters of each solution was treated with 100 μL of 1% solution of l-FDAA in acetone followed by 1.0 M NaHCO_3_ (40 μL). The reaction mixture was incubated for 1 h at 45 °C before being quenched by 1.0 M HCl (40 μL). The derivatives of the hydrolysates and standard amino acids were analyzed by HPLC column (YMC-pack ODS-A, 10 × 250 mm, 5 μm, 1 mL/min) at 30 °C using the following gradient program: solvent A, water + 0.2% TFA; solvent B, MeCN; linear gradient: 0 min 25% B, 40 min 60% B, 45 min 100% B; UV detection at 340 nm. HPLC analysis showed that the retention times for *L*-FDAA derivatives of hydrolysates of **4/5**, standard l-Ala and standard d-Ala were 11.7, 11.7 and 14.3 min, respectively (Figures S26 and S27).

Similarly, Compound **8**, standard l-phenylalanine and d-phenylalanine were also derivatized with l-FDAA and analyzed by HPLC column. HPLC analysis revealed that acid hydrolysates of **8** displayed the same retention time (24.1 min) as the d-phenylalanine but was different from l-phenylalanine (21.8 min) (Figure S28).

### 3.6. Bioassays

#### 3.6.1. Zebrafish Embryos

Zebrafish (*Danio rerio*) of the AB wild-type strain and TG (VEGFR2:GFP) type strain were maintained under a 14 h light/10 h dark cycle in an automatic circulating tank system and fed with artificial granular bait and fresh *Artemia nauplii* [21]. Adult mating pairs were placed in a breeding tank in the evening, and fertilized eggs were collected in the next morning (9 h). After disinfected, fertilized eggs raised in culture solution (5.0 mM NaCl, 0.17 mM KCl, 0.4 mM CaCl_2_, and 0.16 mM MgSO_4_) in a light-operated incubator at 28.0 °C ± 0.5 °C [22].

#### 3.6.2. Heart Rate Experiments

Zebrafish embryos cultured for 48 h were arrayed into 24-well microtiter plates (six to eight embryos and 1000 μL culture solution per well). Each test compound was diluted with DMSO, and 1 μL of the working stock was added to each well, resulting in a final screening concentration of 1, 10 and 100 μg/mL, respectively. Then 2 μM ASM was added to all model groups. Two micromols of ASM was used as positive control and 1 ‰ DMSO was tested as negative control. After 24 h incubation with test compounds, the heart rate of each zebrafish was recorded by the inverted microscope (XSJ-D) (Figure 8) [10].

#### 3.6.3. Vasculogenesis Experiments

Egg membranes were removed from embryos by pronase E solution (1.0 mg/mL) (Shanghai, China) at 24 h post fertilization. Then zebrafish embryos were added to 24-well microtiter plates (six to eight embryos per well) treated with 20, 50 and 100 μg/mL of each test compound and 4 μg/mL of vatalanib (PTK787, Basel, Switzerland). The positive control was 4 μg/mL PTK787 and the negative control was 1‰ DMSO (Shanghai, China). Before arraying the plate, unhealthy or developmentally delayed embryos were removed by examination under a stereoscopic microscope. After 24 h incubation in a light-operated incubator at 28.0 °C ± 0.5 °C, the number and length of intersegmental vessels were captured using a fluorescent microscope (SZX16 Tokyo, Japan) or image acquisition systems (DP2-BSW, Tokyo, Japan) (Figure 9) [10].

#### 3.6.4. Antiangiogenic Vessel Growth Experiments

Zebrafish embryos cultured for 24 h were placed in 24-well microtiter plates, with six to eight embryos and 1000 μL culture solution per well. Embryos were exposed to the compound solutions at 1, 10 and 100 μg/mL. At 48 hpf (hours post fertilization), the influence on antiangiogenic vessel growth of each zebrafish was observed using inverted microscope (XSJ-D, Chongqing, China) and an automated custom algorithm was used to quantify the number of angiogenic vessels in the zebrafish.

## 4. Conclusions

Three new alkaloids (**1**, **4** and **8**), together with nine known analogues (**2**, **3**, **5**–**7**, and **9**–**12**), were isolated from the marine-derived fungus *Penicillium expansum* Y32. Their structures including the absolute configurations were elucidated by spectroscopic and Mosher’s and Marfey’s methods, along with quantum ECD calculations. Each of the compounds was evaluated for cardiovascular effects with live a zebrafish model. All of the compounds showed significant mitigative effects on bradycardia caused by astemizole (ASM) in the heart rate experiments. Compounds **4**–**6** and **8**–**12** exhibited potent vasculogenetic activity in the vasculogenesis experiments. The results suggested that these compounds could be promising candidates for cardiovascular disease lead compounds.

## Supplementary Files

Supplementary File 1

## References

[1] World Health Organization Cardiovascular diseases (CVDs). http://www.who.int/mediacentre/factsheets/fs317/en/.

[2] Hideo H., Hirotaka M., Kohki A. (2004). New insecticidal compounds, communesins C, D and E, from *Penicillium expansum* link MK-57. Biosci. Biotechnol. Biochem..

[3] Dalsgaard P.W., Blunt J.W., Munro M.H.G., Frisvad J.C., Christophersen C. (2005). Communesins G and H, new alkaloids from the psychrotolerant fungus *Penicillium rivulum*. J. Nat. Prod..

[4] Zuo Z.W., Ma D. (2011). Enantioselective Total Syntheses of Communesins A and B. Angew. Chem. Int. Ed..

[5] Fremlin L.J., Piggott A.M., Lacey E., Capon R.J. (2009). Cottoquinazoline A and Cotteslosins A and B, metabolites from an Australian marine-derived strain of *Aspergillus versicolor*. J. Nat. Prod..

[6] Peng J.X., Lin T., Wang W., Xin Z.H., Zhu T.J., Gu Q.Q., Li D.H. (2013). Antiviral alkaloids produced by the mangrove-derived fungus *Cladosporium* sp. PJX-41. J. Nat. Prod..

[7] Lee S.U., Asami Y., Lee D., Jang J.H., Ahn J.S., Oh H. (2011). Protuboxepins A and B and protubonines A and B from the marine-derived fungus *Aspergillus* sp. SF-5044. J. Nat. Prod..

[8] Oikawa H., Murakami Y., Ichihara A. (1993). 20-Ketoreductase Activity of Chaetoglobosin A and prochaetoglobosins in a cell-free system of chaetomium subaffine and the isolation of new chaetoglobosins. Biosci. Biotech. Biochem..

[9] Iwamoto C., Yamada T., Ito Y., Minoura K., Numata A. (2001). Cytotoxic cytochalasans from a *Penicillium* species separated from a marine alga. Tetrahedron.

[10] Tran T.C., Sneed B., Haider J., Blavo D., White A., Aiyejorun T., Baranowski T.C., Rubinstein A.L., Doan T.N., Dingledine R. (2007). Automated, quantitative screening assay for antiangiogenic compounds using transgenic zebrafish. Cancer Res..

[11] Matsumori N., Kaneno D., Murata M., Nakamura H., Tachibana K. (1999). Stereochemical determination of acyclic structures based on carbon-proton spin-coupling constants. A method of configuration analysis for natural products. J. Org. Chem..

[12] Frisch M.J., Trucks G.W., Schlegel H.B., Scuseria G.E., Robb M.A., Cheeseman J.R., Montgomery J.A., Vreven T., Kudin K.N., Burant J.C. (2004). Gaussian 03, Revision E.01.

[13] Ueoka R., Nakao Y., Kawatsu S., Yaegashi J., Matsumoto Y., Matsunaga S., Furihata K., van Soest R.W., Fusetani N. (2009). Gracilioethers A–C, antimalarial metabolites from the marine sponge *Agelas gracilis*. J. Org. Chem..

[14] Zhuang Y.B., Teng X.C., Wang Y., Liu P.P., Li G.Q., Zhu W.M. (2011). New quinazolinone alkaloids within rare amino acid residue from coral-associated fungus, *Aspergillus versicolor* LCJ-5-4. Org. Lett..

[15] Takahashi C., Matsushita T., Doi M., Minoura K., Shingu T., Kumeda Y., Numata A. (1995). Fumiquinazolines A–G, novel metabolites of a fungus separated from a *Pseudolabrus* marine fish. J. Chem. Soc. Perkin Trans..

[16] Marfey P. (1984). Determination of d-amino acids. II. Use of a bifunctional reagent, 1,5-difluoro-2,4-dinitrobenzene. Carlsberg Res. Commum..

[17] Li G.Y., Li L.M., Yang T., Chen X.Z., Fang D.M., Zhang G.L. (2010). Four new alkaloids, brevianamides O–R, from the fungus *Aspergillus versicolor*. Helv. Chim. Acta.

[18] Chan J., Bayliss P.E., Wood J.M., Roberts T.M. (2002). Dissection of angiogenic signaling in zebrafish using a chemical genetic approach. Cancer Cell.

[19] Cross L.M., Cook M.A., Lin S., Chen J.N., Rubinstein A.L. (2003). Rapid analysis of angiogenesis drugs in a live fluorescent zebrafish assay. Arterioscler. Thromb. Vasc. Biol..

[20] Seng W.L., Eng K., Lee J., McGrath P. (2004). Use of a monoclonal antibody specific for activated endothelial cells to quantitate angiogenesis *in vivo* in zebrafish after drug treatment. Angiogenesis.

[21] Han Y., Zhang J.P., Qian J.Q., Hu C.Q. (2015). Cardiotoxicity evaluation of anthracyclines in zebrafish (*Danio rerio*). J. Appl. Toxicol..

[22] McKinley E.T., Baranowski T.C., Blavo D.O., Cato C., Doan T.N., Rubinstein A.L. (2005). Neuroprotection of MPTP-induced toxicity in zebrafish dopaminergic neurons. Mol. Brain Res..

