# Supplementary material for: Alkaloids with Cardiovascular Effects from the Marine-Derived Fungus Penicillium expansum Y32
Source: Mar Drugs. 2015 Oct 22;13(10):6489–504. doi: 10.3390/md13106489 (PMC4626702; doi:10.3390/md13106489)
Supplement: Supplementary File 1 [file marinedrugs-13-06489-s001.docx]

Supplementary Materials

List of Supplementary Materials

**The 18S rRNA sequences data of *Penicillium expansum* Y32**…………………………………S2

**Figure S1.** ^1^H-NMR spectrum of camemberol I (**1**) in CDCl_3_………………………………………S3

**Figure S2.** ^13^C-NMR spectrum of camemberol I (**1**) in CDCl_3_……………………………………S4

**Figure S3.** DEPT spectrum of camemberol I (**1**) in CDCl_3_…………………………………………S5

**Figure S4.** HSQC spectrum of camemberol I (**1**) in CDCl_3_………………………………………S6

**Figure S5.** ^1^H-^1^H COSY spectrum of camemberol I (**1**) in CDCl_3_…………………………………S7

**Figure S6.** HMBC spectrum of camemberol I (**1**) in CDCl_3_………………………………………S8

**Figure S7.** NOESY spectrum of camemberol I (**1**) in CDCl_3_………………………………………S9

**Figure S8.** ^1^H-NMR spectrum of fumiquinazoline Q (**4**) in CDCl_3_………………………………S10

**Figure S9.** ^13^C-NMR spectrum of fumiquinazoline Q (**4**) in CDCl_3_………………………………S11

**Figure S10.** DEPT spectrum of fumiquinazoline Q (**4**) in CDCl_3_…………………………………S12

**Figure S11.** HSQC spectrum of fumiquinazoline Q (**4**) in CDCl_3_…………………………………S13

**Figure S12.** ^1^H-^1^H COSY spectrum of fumiquinazoline Q (**4**) in CDCl_3_…………………………S14

**Figure S13.** HMBC spectrum of fumiquinazoline Q (**4**) in CDCl_3_………………………………S15

**Figure S14.** NOESY spectrum of fumiquinazoline Q (**4**) in CDCl_3_………………………………S16

**Figure S15.** ^1^H-NMR spectrum of protuboxepin E (**8**) in DMSO-*d*_6_………………………………S17

**Figure S16.** ^13^C-NMR spectrum of protuboxepin E (**8**) in DMSO-*d*_6_……………………………S18

**Figure S17.** DEPT spectrum of protuboxepin E (**8**) in DMSO-*d*_6_…………………………………S19

**Figure S18.** HSQC spectrum of protuboxepin E (**8**) in DMSO-*d*_6_………………………………S20

**Figure S19.** ^1^H-^1^H COSY spectrum of protuboxepin E (**8**) in DMSO-*d*_6_…………………………S21

**Figure S20.** HMBC spectrum of protuboxepin E (**8**) in DMSO-*d*_6_……………………...……S22

**Figure S21.** NOESY spectrum of protuboxepin E (**8**) in DMSO-*d*_6_………………………………S23

**Figure S22.** ^1^H-NMR spectrum of (*S*)-**1** MTPA ester (**1a**) in CDCl_3_………………………………S24

**Figure S23.** ^1^H-^1^H COSY spectrum of (*S*)-**1** MTPA ester (**1a**) in CDCl_3_…………………………S25

**Figure S24.** ^1^H-NMR spectrum of (*R*)-**1** MTPA ester (**1b**) in CDCl_3_………………………………S26

**Figure S25.** ^1^H-^1^H COSY spectrum of (*R*)-**1** MTPA ester (**1b**) in CDCl_3_…………………………S27

**Figure S26.** Marfey’s method applying for compound **4**…………………………………………S29

**Figure S27.** Marfey’s method applying for compound **5**…………………………………………S30

**Figure S28.** Marfey’s method applying for compound **8**…………………………………………S31

**Table S1.** ^1^H (600 MHz) and ^13^C (150 MHz) NMR data for compounds **2**–**3** in CDCl_3_ ………S32

The 18S rRNA Sequences Data of *Penicillium expansum* Y32

AACTTGTACTGTGAAACTGCGAATGGCTCATTAAATCAGTTATCGTTTATTTGATAGTACCTTACTACATGGATACCTGTGGTAATTCTAGAGCTAATACATGCTAAAAACCCCGACTTCAGGAAGGGGTGTATTTATTAGATAAAAAACCAACGCCCTTCGGGGCTCCTTGGTGAATCATAATAACTTAACGAATCGCATGGCCTTGCGCCGGCGATGGTTCATTCAAATTTCTGCCCTATCAACTTTCGATGGTAGGATAGTGGCCTACCATGGTGGCAACGGGTAACGGGGAATTAGGGTTCGATTCCGGAGAGGGAGCCTGAGAAACGGCTACCACATCCAAGGAAGGCAGCAGGCGCGCAAATTACCCAATCCCGATACGGGGAGGTAGTGACAATAAATACTGATACGGGGCTCTTTTGGGTCTCGTAATTGGAATGAGAACAATTTAAATCCCTTAACGAGGAACAATTGGAGGGCAAGTCTGGTGCCAGCAGCCGCGGTAATTCCAGCTCCAATAGCGTATATTAAAGTTGTTGCAGTTAAAAAGCTCGTAGTTGAACCTTGGGTCTGGCTGGCCGGTCCGCCTCACCGCGAGTACTGGTCCGGCTGGACCTTTCCTTCTGGGGAACCTCATGGCCTTCACTGGCTGTGGGGGGAACCAGGACTTTTACTGTGAAAAAATTAGAGTGTTCAAAGCAGGCCTTTGCTCGAATACATTAGCATGGAATAATAGAATAGGACGTGTGGTTCTATTTTGTTGGTTTCTAGGACCGCCGTAATGATTAATAGGGATAGTCGGGGGCGTCAGTATTCAGCTGTCAGAGGTGAAATTCTTGGATTTGCTGAAGACTAACTACTGCGAAAGCATTCGCCAAGGATGTTTTCATTAATCAGGGAACGAAAGTTAGGGGATCGAAGACGATCAGATACCGTCGTAGTCTTAACCATAAACTATGCCGACTAGGGATCGGACGGGATTCTATAATGACCCGTTCGGCACCTTACGAGAAATCAAAGTTTTTGGGTTCTGGGGGGAGTATGGTCGCAAGGCTGAAACTTAAAGAAATTGACGGAAGGGCACCACAAGGCGTGGAGCCTGCGGCTTAATTTGACTCAACACGGGGAAACTCACCAGGTCCAGACAAAATAAGGATTGACAGATTGAGAGCTCTTTCTTGATCTTTTGGATGGTGGTGCATGGCCGTTCTTAGTTGGTGGAGTGATTTGTCTGCTTAATTGCGATAACGAACGAGACCTCGGCCCTTAAATAGCCCGGTCCGCATTTGCGGGCCGCTGGCTTCTTAAGGGGACTATCGGCTCAAGCCGATGGAAGTGCGCGGCAATAACAGGTCTGTGATGCCCTTAGATGTTCTGGGCCGCACGCGCGCTACACTGACAGGGCCAGCGAGTACATCACCTTAACCGAGAGGTTTGGGTAATCTTGTTAAACCCTGTCGTGCTGGGGATAGAGCATTGCAATTATTGCTCTTCAACGAGGAATGCCTAGTAGGCACGAGTCATCAGCTCGTGCCGATTACGTCCCTGCCCTTTGTACACACCGCCCGTCGCTACTACCGATTGAATGGCTCAGTGAGGCCTTGGGATTGGCTTAGGAGGGTTGGCAACGACCCCCCAGAGCCGAAAACTTGGTCAAACTCGGTCA

**Figure S1.** ^1^H-NMR spectrum of camemberol I (**1**) in CDCl_3_.

**Figure S2.** ^13^C-NMR spectrum of camemberol I (**1**) in CDCl_3_.

**Figure S3.** DEPT spectrum of camemberol I (**1**) in CDCl_3_.

**Figure S4.** HSQC spectrum of camemberol I (**1**) in CDCl_3_.

**Figure S5.** ^1^H-^1^H COSY spectrum of camemberol I (**1**) in CDCl_3_.

**Figure S6.** HMBC spectrum of camemberol I (**1**) in CDCl_3_.

**Figure S7.** NOESY spectrum of camemberol I (**1**) in CDCl_3_.

**Figure S8.** ^1^H-NMR spectrum of fumiquinazoline Q (**4**) in CDCl_3_.

**Figure S9.** ^13^C-NMR spectrum of fumiquinazoline Q (**4**) in CDCl_3_.

**Figure S10.** DEPT spectrum of fumiquinazoline Q (**4**) in CDCl_3_.

**Figure S11.** HSQC spectrum of fumiquinazoline Q (**4**) in CDCl_3_.

**Figure S12.** ^1^H-^1^H COSY spectrum of fumiquinazoline Q (**4**) in CDCl_3_.

**Figure S13.** HMBC spectrum of fumiquinazoline Q (**4**) in CDCl_3_.

**Figure S14.** NOESY spectrum of fumiquinazoline Q (**4**) in CDCl_3_.

**Figure S15.** ^1^H-NMR spectrum of protuboxepin E (**8**) in DMSO-*d*_6_.

**Figure S16.** ^13^C-NMR spectrum of protuboxepin E (**8**) in DMSO-*d*_6_.

**Figure S17.** DEPT spectrum of protuboxepin E (**8**) in DMSO-*d*_6_.

**Figure S18.** HSQC spectrum of protuboxepin E (**8**) in DMSO-*d*_6_.

**Figure S19.** ^1^H-^1^H COSY spectrum of protuboxepin E (**8**) in DMSO-*d*_6_.

**Figure S20.** HMBC spectrum of protuboxepin E (**8**) in DMSO-*d*_6_.

**Figure S21.** NOESY spectrum of protuboxepin E (**8**) in DMSO-*d*_6_.

**Figure S22.** ^1^H-NMR spectrum of (*S*)-**1** MTPA ester (**1a**) in CDCl_3_.

**Figure S23.** ^1^H-^1^H COSY spectrum of (*S*)-**1** MTPA ester (**1a**) in CDCl_3_.

**Figure S24.** ^1^H-NMR spectrum of (*R*)-**1** MTPA ester (**1b**) in CDCl_3_.

**Figure S25.** ^1^H-^1^H COSY spectrum of (*R*)-**1** MTPA ester (**1b**) in CDCl_3_.

(**a**)

(**b**)

(**c**)

**Figure S26.** *Cont.*

(**d**)

**Figure S26.** Marfey’s method applying for compound **4** (ODS column: Solvent A: water
+ 0.2% TFA; Solvent B: MeCN; linear gradient: 0 min 25% B, 40 min 60% B, 45 min 100% B; Flow rate: 1 mL/min; Teperature, 30 °C; UV detection at 340 nm; FDAA,
13.6 min). (**a**) Co-injection of FDAA derivatives of standard D-Ala by ODS column;
(**b**) Co-injection of FDAA derivatives of standard L-Ala by ODS column; (**c**) FDAA derivatives of the acid hydrolysate of **4** by ODS column; (**d**) Co-injection of FDAA derivatives of the acid hydrolysate of **4** with standard L-Ala by ODS column.

(**a**)

(**b**)

**Figure S27.** *Cont.*

(**c**)

(**d**)

**Figure S27.** Marfey’s method applying for compound **5** (ODS column: Solvent A: water
+ 0.2% TFA; Solvent B: MeCN; linear gradient: 0 min 25% B, 40 min 60% B, 45 min 100% B; Flow rate: 1 mL/min; Teperature, 30 °C; UV detection at 340 nm; FDAA, 13.6 min).
(**a**) Co-injection of FDAA derivatives of standard D-Ala by ODS column; (**b**) Co-injection of FDAA derivatives of standard L-Ala by ODS column; (**c**) FDAA derivatives of the acid hydrolysate of **5** by ODS column; (**d**) Co-injection of FDAA derivatives of the acid hydrolysate of **5** with standard L-Ala by ODS column.

(**a**)

**Figure S28.** *Cont.*

(**b**)

(**c**)

(**d**)

**Figure S28.** Marfey’s method applying for compound **8** (ODS column: Solvent A: water
+ 0.2% TFA; Solvent B: MeCN; linear gradient: 0 min 25% B, 40 min 60% B, 45 min
100% B; Flow rate: 1 mL/min; Teperature, 30 °C; UV detection at 340 nm; FDAA,
13.6 min). (**a**) Co-injection of FDAA derivatives of standard l-phenylalanine by
ODS column; (**b**) Co-injection of FDAA derivatives of standard d-phenylalanine by
ODS column; (**c**) FDAA derivatives of the acid hydrolysate of **8** by ODS column;
(**d**) Co-injection of FDAA derivatives of the acid hydrolysate of **8** with standard
d-phenylalanine by ODS column.

**Table S1.** ^1^H (600 MHz) and ^13^C (150 MHz) NMR data for compounds **2**–**3** in CDCl_3_.

| **2** | | **3** | |
| --- | --- | --- | --- |
| **δ_C_** | **δ_H_, Mult. (*J* in Hz)** | **δ_C_** | **δ_H_, Mult. (*J* in Hz)** |
| 123.3, CH | 6.68, t (7.6) | 123.4, CH | 6.64, t (7.6) |
| 120.7, CH | 6.69, t (7.7) | 120.5, CH | 6.68, t (7.7) |
| 127.6, CH | 7.00, t (7.6) | 127.4, CH | 7.00, d (7.6) |
| 117.1, CH | 6.71, t (7.6) | 116.9, CH | 6.64, d (7.6) |
| 142.8, C |  | 142.8, C |  |
| 82.6, CH | 4.69, s | 82.4, CH | 4.70, s |
| 51.6, C |  | 51.4, C |  |
| 132.4, C |  | 132.4, C |  |
| 52.1, C |  | 52.2, C |  |
| 132.5, C |  | 132.3, C |  |
| 79.8, CH | 5.02, s | 79.1, CH | 5.11, s |
| 65.5, CH | 4.08, d (9.0) | 65.6, CH | 4.18, d (9.0) |
| 136.9, C |  | 136.6, C |  |
| 113.3, CH | 6.06, d (7.7) | 113.2, CH | 6.08, d (7.6) |
| 129.0, CH | 6.88, t (7.7) | 128.9, CH | 6.88, t (7.7) |
| 102.0, CH | 5.95, d (7.6) | 101.9, CH | 5.94, d (7.7) |
| 150.7, C |  | 150.6, C |  |
| 44.2, CH_2_ | 3.47, dd (11.6, 8.6);  3.36, dd (11.8, 8.7) | 44.3, CH_2_ | 3.87, dd (11.9, 8.4);  3.07, dd (11.9, 8.5) |
| 30.9, CH_2_ | 2.27, dt (13.1, 7.3);  1.97, dd (13.2, 7.2) | 30.5, CH_2_ | 2.71, dd (13.5, 7.7);  1.99, dd (13.1, 7.3) |
| 38.2, CH_2_ | 2.74, dt (12.5, 8.3);  2.36, dd (12.8, 8.4) | 37.9, CH_2_ | 2.36, dt (12.2, 8.5);  2.27, dt (12.7, 8.2) |
| 36.4, CH_2_ | 3.89, dd (11.5, 8.9);  3.01, dt (11.5, 8.4) | 36.1, CH_2_ | 3.48, dd (15.8, 8.6);  3.4, dd (15.8, 8.7) |
| 64.2, CH | 2.87, d (9.0) | 64.0, CH | 2.90, d (9.0) |
| 59.9, C |  | 59.8, C |  |
| 20.6, CH_3_ | 1.38, s | 25.0, CH_3_ | 1.42, s |
| 24.9, CH_3_ | 1.53, s | 20.6, CH_3_ | 1.65, s |
| 29.8, CH_3_ | 2.84, s | 29.7, CH_3_ | 2.84, s |
| 172.1, C |  | 168.5, C |  |
| 22.8, CH_3_ | 2.33, s | 121.4, CH | 6.56, d (15.1) |
|  |  | 141.9, CH | 7.31, dd (15.1, 10.8) |
|  |  | 130.8, CH | 6.19, m |
|  |  | 137.2, CH | 6.10, d (6.7) |
|  |  | 18.8, CH_3_ | 1.84, d (6.7) |

*Communesin A* (**2**): White amorphous powder; [α] −29 (*c* 0.1, MeOH); UV (MeOH) λ_max_ 206 (3.74), 248 (3.47), 268 (3.17), 316 (2.65) nm; CD (*c* 0.1, MeOH) λ_max_ (Δ*ε*) 218 (−1.4), 244 (+2.0), 270 (−2.2), 294 (+1.8), 319 (−1.0) nm; ^1^H and ^13^C NMR data, see Table 1. HRESIMS *m*/*z* 457.2607
[M + H]^+^ (calcd for C_28_H_33_N_4_O_2_, 457.2604).

*Communesin B* (**3**): White amorphous powder; [α] −69 (*c* 0.1, MeOH); UV (MeOH) λ_max_ 206 (3.93), 268 (3.85) nm; CD (*c* 0.1, MeOH) λ_max_ (Δ*ε*) 216 (−2.2), 245 (+2.0), 270 (−2.3), 294 (+2.1), 319 (−0.1) nm; ^1^H and ^13^C NMR data, see Table 1. HRESIMS *m*/*z* 509.2919 [M + H]^+^ (calcd for C_32_H_37_N_4_O_2_, 509.2917).
